# Entry of *Francisella tularensis* into Murine B Cells: The Role of B Cell Receptors and Complement Receptors

**DOI:** 10.1371/journal.pone.0132571

**Published:** 2015-07-10

**Authors:** Lenka Plzakova, Zuzana Krocova, Klara Kubelkova, Ales Macela

**Affiliations:** Department of Molecular Pathology and Biology, Faculty of Military Health Sciences, University of Defence, Hradec Kralove, Czech Republic; University of Louisville, UNITED STATES

## Abstract

*Francisella tularensis*, the etiological agent of tularemia, is an intracellular pathogen that dominantly infects and proliferates inside phagocytic cells but can be seen also in non-phagocytic cells, including B cells. Although protective immunity is known to be almost exclusively associated with the type 1 pathway of cellular immunity, a significant role of B cells in immune responses already has been demonstrated. Whether their role is associated with antibody-dependent or antibody-independent B cell functions is not yet fully understood. The character of early events during B cell–pathogen interaction may determine the type of B cell response regulating the induction of adaptive immunity. We used fluorescence microscopy and flow cytometry to identify the basic requirements for the entry of *F*. *tularensis* into B cells within *in vivo* and *in vitro* infection models. Here, we present data showing that *Francisella tularensis* subsp. *holarctica* strain LVS significantly infects individual subsets of murine peritoneal B cells early after infection. Depending on a given B cell subset, uptake of *Francisella* into B cells is mediated by B cell receptors (BCRs) with or without complement receptor CR1/2. However, *F*. *tularensis* strain FSC200 Δ*iglC* and Δ*ftdsbA* deletion mutants are defective in the ability to enter B cells. Once internalized into B cells, *F*. *tularensis* LVS intracellular trafficking occurs along the endosomal pathway, albeit without significant multiplication. The results strongly suggest that BCRs alone within the B-1a subset can ensure the internalization process while the BCRs on B-1b and B-2 cells need co-signaling from the co receptor containing CR1/2 to initiate *F*. *tularensis* engulfment. In this case, fluidity of the surface cell membrane is a prerequisite for the bacteria’s internalization. The results substantially underline the functional heterogeneity of B cell subsets in relation to *F*. *tularensis*.

## Introduction

General characteristics of bacterial intracellular pathogens, such as *Brucellae*, *Listeriae*, *Salmonellae*, and *Francisellae*, include their adhesion to, internalization into, and proliferation within professional phagocytes. Macrophages are considered to be the preferred target. In addition to infecting professional phagocytes, however, intracellular bacteria also attack non-phagocytic cells. *Brucellae*, for example, are able to invade a broad range of mammalian cell lines [1–3] as well as non-phagocytic cells *in vivo*, including erythrocytes [4]. *Salmonella* enters the spectrum of non-phagocytic eukaryotic cells using the so-called “trigger” mechanism induced by a specialized secretory apparatus–the Type III secretion system (for review, see [5]). *Listeria monocytogenes*, using proteins InlA and InlB, which interact with cellular E-cadherin (an adhesion protein) and Met (also known as hepatocyte growth factor [HGF] receptor), respectively, generally activates a zipper-like mechanism for entry into the eukaryotic cell (for review, see [6–9].

*Francisella tularensis*, the causative agent of tularemia, is a facultative intracellular pathogen causing zoonotic disease in a wide variety of species, ranging from protozoa to vertebrates, including humans [10–12]. An intracellular pathogen with transient extracellular phase [13], it dominantly infects and proliferates inside phagocytic cell types both *in vitro* and *in vivo* [14–17]. Like other intracellular pathogens, *Francisellae* can be found also within non-phagocytic cells. Lung macrophages and dendritic cells as well as lung endothelial cells and structural alveolar type II epithelial cells are infected in the course of the pneumonic form of tularemia [18]. *Francisella* uptake has been documented in hepatocyte cell lines [19], fibroblasts, various epithelial cell lines, endothelial cells [20], and even erythrocytes [21].

In general, the first steps in the bacterial cell invasion process are recognition of the host cell and the bacteria’s attachment to it. As the recognition of *Francisellae* by TLR2 is a critical point in the host’s protective response [22,23], attachment is a critical element in the process of bacteria internalization. *Francisella* exposes several proteins at the outer membrane that probably ensure close interaction of the bacterium with the host cell. There is evidence that type IV pili [24], *Francisella* outer membrane protein FsaP [25], or *Francisella* elongation factor-Tu [26] ensure adherence of the bacterium to a host cell under nonopsonic conditions. Under opsonic conditions, the “bridges”between cell membranes ensure the presence of opsonins, as are for example components of a complete serum or surfactants, which effectively mediate the internalization of *Francisellae* into host cells.

Internalization alone from the side of the host cell can be mediated by different cell surface receptors depending upon the conditions under which the process is occurring. Actin rearrangement and active microtubules finalize the internalization process [27,28]. Uptake of nonopsonized bacteria by macrophages seems to be mediated dominantly by the mannose receptor [29]. The complement receptors (CRs) CR1/2, CR3, and CR4 as well as macrophage scavenger receptor class A have been shown, under specific conditions, to participate in the uptake of complete serum-opsonized *F*. *tularensis* into the various professional phagocytes. *Francisellae* opsonized by antibodies are almost exclusively internalized through ligation of Fc receptors (for review, see [12]). Moreover, the opsonization of *Francisellae* and their redirection from the mannose receptor to Fc receptors and CRs lead to substantial modulation of intracellular trafficking and the final fate of bacteria inside the phagocyte. Opsonophagocytic receptors alter the intracellular fate of *Francisella* by delivering bacteria through phagocytic pathways that restrict phagosomal escape and intracellular proliferation [30].

Recently, we documented that *Francisella* infect *in vitro* murine and human B cell lines and *in vivo* murine peritoneal as well as spleen B cells [31,32]. Both human and mice B cells have been shown to bind various species of bacteria, including such intracellular bacteria as *Brucella* [33,34], *Mycobacterium tuberculosis* [35], and *Chlamydia trachomatis* [36]. Internalization into B cells of one of the *Brucella* subtypes, *Brucella abortus*, recently was shown to be promoted by functional microfilaments. Once inside the B cell, the *Brucella* resides in a late-endosomal/lysosomal compartment [37]. Similarly, *Salmonella enterica* serovar Typhimurium invades B cells. The living niche there is the *Salmonella*-containing vacuole (SCV), which can be characterized as a late-endosomal/lysosomal compartment [38]. The invasion of *Salmonella* into B cells is an active process controlled by SPI-1, which induces ruffling of the B cell surface membrane that is followed by macropinocytosis of the bacteria and creation of the spacious SCV [39]. There is evidence that B cell receptor (BCR) is engaged in the uptake of *Salmonellae* into B cells. BCR-mediated internalization of *Salmonellae* into B cells significantly modulates the fate of the bacterium inside the B cell as well as the fate of the B cell itself. *Salmonella* exists inside B cells in a non-replicative state. The infected B cells undergo differentiation instead of apoptosis, and they secrete anti-*Salmonella* antibodies [40,41].

Here, we demonstrate that *Francisella tularensis* subsp. *holarctica* strain LVS (FSC155) significantly infects subtypes of murine peritoneal B cells early after intraperitoneal infection. The uptake of *Francisella* into B cells is mediated by the BCR and CRs.

## Materials and Methods

### Bacteria and growth conditions

*Francisella tularensis* LVS (FSC155), live vaccine strain, and *F*. *tularensis* LVS/GPF expressing green fluorescent protein (bacteria were kindly provided by Åke Forsberg, FOI, Umea, Sweden), *F*. *tularensis DsbA* mutant [42], and *F*. *tularensis iglC* mutant [43] were used for the study. All *F*. *tularensis* strains were cultured on McLeod agar enriched with bovine hemoglobin (Becton Dickinson, San Jose, California, USA) and IsoVitalex (Becton Dickinson) at 36.8°C for 24 h. Following incubation, bacterial colonies were lifted from the plates and resuspended in phosphate buffered saline (PBS) such that the optical density was 1.00 (corresponding to bacterial 5 x 10^9^ CFU/mL). The actual number of bacteria in the suspension utilized for the experiments was determined by serial dilutions (10^−6^ and 10^−7^) and the number of colony-forming units (CFU) was calculated.

### Animals

Female specific pathogen-free BALB/c mice were purchased from Velaz (Unetice, CZ) and were used at 6–8 weeks of age. Mice were placed in sterile cages with an air-conditioner and stabilized temperature 22 ± 2°C. A 12 h light and 12 h dark regime was used. *In vivo* experiments on mice were conducted under supervision of the institution’s Animal Unit and were approved by the Animal Care and Use Committee of the Faculty of Military Health Sciences, University of Defense, Hradec Kralove, Czech Republic under project number 4/10.

### Cell suspension

The mouse B cell line A20 (ATCC TIB-208, Manassas, Virginia, USA), derived from BALB/cAnN mice, and peritoneal cells from BALB/c mice were used. Cell suspensions were resuspended in DMEM cultivation medium supplemented with 10% fetal bovine serum and incubated at 36.8°C in a 5% CO_2_ atmosphere.

### Opsonization of bacteria

The volume of 500 μL of murine fresh serum or 500 μL of murine heat-inactivated anti-*F*. *tularensis* serum (hereinafter referred to as “antibodies”) were added to 4 x 10^9^ bacteria, incubated at 36.8°C for 1 h, washed twice by pre-warmed PBS, resuspended in 1 mL saline, and used for the experiments. Sera diluted to subagglutinating titer were added to 1 mL of bacterial suspension. Heat-inactivated murine fresh serum was used as a control.

### Infection of cells

A20 cells or peritoneal cells (5 x 10^5^ per well) were co-cultivated with unopsonized or opsonized *F*. *tularensis* LVS, *F*. *tularensis* LVS/GFP, or *F*. *tularensis* mutants, in total volume of 0.5 mL per well at multiplicity of infection (MOI) 500. Control A20 cells or peritoneal cells were cultivated without infection. After 3 h incubation at 36.8°C in 5% CO2 atmosphere, cultures were washed using PBS. The proportion of infected cells with *F*. *tularensis* LVS/GFP was examined using flow cytometry. *F*. *tularensis* was stained using rabbit anti-*F*. *tularensis* serum as a primary antibody and goat anti-rabbit antibody conjugated with Alexa Fluor 488 (Becton Dickinson, San Jose, California, USA) as a secondary antibody for fluorescent microscopy. DAPI (Invitrogen, Molecular Probes, Oregon, USA) was used to visualize the nuclei.

### Serum preparation

#### Mouse serum

Anti*-F*. *tularensis* serum was obtained by suspending heat-killed *F*. *tularensis* LVS cells in complete Freund’s adjuvant at a concentration of 10^7^ bacteria/mL. The suspension was used for repeating *s*.*c*. immunization of mice. The serum was harvested 21 d after last immunization dose. The titer of sera was determined using enzyme-linked immunosorbent assay (ELISA).

#### Rabbit serum

Purified immune high-titer anti-*F*. *tularensis* rabbit sera were obtained by repeated immunization of rabbits. The first dose was 10^6^ *F*. *tularensis* LVS in Freund’s adjuvant, the second was administered 3 weeks later with 10^8^ *F*. *tularensis* LVS in saline, and the last immunization was done 5 weeks after the first with dose 10^8^ *F*. *tularensis* LVS in saline. Blood was collected two weeks after the final immunization, serum was purified by ammonium sulphate, and ELISA titer was determined.

Autolysate of *F*. *tularensis* colonies was used for ELISA as antigen coated to flat-bottomed microtiter plates. The reciprocal value of two-fold serial dilution of serum (originally diluted 1 to 10) was used to define a serum titer. Corpuscular, heat-inactivated *F*. *tularensis* antigen was used for determination of agglutinating titer.

### Viability of bacteria inside the B cells

BALB/c mice were infected with unopsonized *F*. *tularensis* LVS/GFP. After 24 h, peritoneal cells were collected, resuspended in DMEM cultivation medium supplemented with 2% fetal bovine serum, then incubated with the antibody CD19^-^Alexa Fluor 647 (rat anti-mouse IgG2a, clone: 6D5; BioLegend, San Diego, California, USA). Peritoneal CD19^+^ GFP^+^ cells were sorted using a BD FACSAria II Cell Sorter. The relative purity of the sorted CD19^+^GFP^+^ cells infected with with nonopsonized bacteria measured by flow cytometry was 100% while the relative purity of the sorted CD19^+^GFP^+^ cells ingested with opsonized bacteria was 99.5%. Sorted CD19^+^ GFP^+^ cells were washed using PBS and lysed with 0.1% sodium deoxycholate after washing. Actual numbers of bacteria were determined by serial dilution (10^0^ and 10^−2^) and the number of CFU was calculated.

### B cell subset identification

Peritoneal cells were subjected to flow cytometry analysis using the antibodies CD19-Alexa Fluor 647 (rat anti-mouse IgG2a, clone: 6D5; BioLegend, San Diego, California, USA), CD5-PerCP (rat anti-mouse IgG2a, clone: 53–7.3; BD Pharmingen, San Jose, California, USA), and CD11b-PE (rat anti-mouse IgG2b, clone: M1/70; BD Pharmingen, San Jose, California, USA). Measurement was made using a CyAn ADP flow cytometer (Dako, Glostrup, Denmark). B-1a cells were characterized as CD19^+^CD5^+^CD11b^+^, B-1b cells as CD19^+^CD5^-^CD11b^+^, and B-2 cells as CD19^+^CD5^-^CD11b^-^, using the Cyan ADP flow cytometer and Summit software version 4.3 (Dako, Glostrup, Denmark). Rat IgG2a FITC-conjugated isotype control (Pierce, Thermo Scientific, USA) and rat IgG2b FITC-conjugated isotype control (Pierce, Thermo Scientific, USA) were used as the isotype controls.

### Blocking of the receptors

Peritoneal cells from BALB/c mice were washed using cell wash (PBS with 5% gelatin and 10% sodium azide). Cell suspensions were incubated for 30 min with the following purified blocking antibodies: IgM (for blocking BCR; rat anti-mouse, clone: RMM-1; BioLegend, San Diego, California, USA), CD21/CD35 (for blocking CR1/2; rat anti-mouse, clone: 7G6; BD Pharmingen, San Jose, California, USA), CD11b (for blocking CR3; rat anti-mouse, clone: M1/70; BD Pharmingen), CD11c (for blocking CR4; Armenian hamster anti-mouse, clone: N418; BD Pharmingen), and FcγIII/II (CD16/32 for blocking FcR; rat anti-mouse, clone: 2.4G2; BD Pharmingen). To block unspecific binds, cells were treated with normal rat IgG isotype control (RD System, USA) or Armenian hamster IgG isotype control (Pierce, Thermo Scientific, USA) before staining. Cell suspensions were then washed three times with PBS and infected as described.

### Membrane rafts disturbance

To reveal the role of the cholesterol-rich membrane domains in internalizing *F*. *tularensis* into peritoneal B cells, cells were treated with 10 μg/mL filipin (Sigma-Aldrich, St. Louis, Missouri, USA) or 10 mM methyl-β-cyclodextrin (Sigma-Aldrich) for 30 min. Finally, flow cytometry was used to enumerate the infected B cell percentage in the cultures.

### Intracellular trafficking of *Francisella* inside the B cells

Fluorescence microscopy was used to identify the colocalization of bacteria and endosomal and lysosomal markers. A20 cells, at total volume 0.5 mL (1 x 10^6^ per well), were infected with *F*. *tularensis* LVS at MOI 500. Infected cells were incubated in DMEM in 5% CO_2_ at 37°C for 5, 15 and 30 min, as well as 1 and 2 h, then washed twice in PBS. The suspensions of cells were adhered to microscope slides using cytospin. The cells were fixed with 3.8% formaldehyde for 30 min. After fixation, cells were washed three times in PBS, neutralized by incubation in PBS with 50 mM NH_4_Cl for 10 min, permeabilized by 0.1% Triton X-100 for 5 min, and blocked by 3% bovine serum albumin (BSA) in PBS for 60 min at room temperature.

Staining: cells were incubated for 45 min with the primary antibody against *F*. *tularensis*–purified immune high-titer anti-*F*. *tularensis* rabbit serum (subagglutinating ELISA titer 1:12800) diluted 1:3000 in 3% BSA in PBS and subsequently with secondary antibody goat anti-rabbit Alexa Fluor 488 (Molecular Probes, Grand Island, New York, USA) diluted 1:500 in 3% BSA in PBS. To detect LAMP-1, cells were stained with rat anti-mouse LAMP-1 antibody (Santa Cruz, Dallas, Texas, USA) diluted 1:100 in 3% BSA in PBS and then with secondary antibody donkey anti-rat Alexa Fluor 594 (Molecular Probes) diluted 1:500 in 3% BSA in PBS. Used for staining EEA1 were goat polyclonal anti-mouse EEA1 (Santa Cruz) diluted 1:100 in 3% BSA in PBS as primary antibody and donkey anti-goat Alexa Fluor 594 (Molecular Probes) diluted 1:500 in 3% BSA in PBS as secondary antibody. To detect Cathepsin D, goat polyclonal anti-mouse Cathepsin D (Santa Cruz) diluted 1:200 in 3% BSA in PBS was used as primary antibody and donkey anti-goat Alexa Fluor 594 (Molecular Probes) diluted 1:500 in 3% BSA in PBS as secondary antibody. Cells were incubated with all antibodies for 45 min, washed three times with PBS, then mounted using Mowiol (Sigma-Aldrich). Microscope slides were viewed by fluorescence microscopy on a Nikon Eclipse Ti (Nikon, Tokyo, Japan). Percentages of colocalization were calculated for an average of 200 cross-sectional images per time point.

### Statistical analysis

We carried out all experiments at least three times, with each sample of an individual cell suspension being cultivated in triplicate. An unpaired Student’s two-tailed *t*-test was applied to all measured data to evaluate statistical significance. *P* values < 0.05 were accepted as significantly different and were denoted by asterisks.

## Results

### *Francisella tularensis* infects subsets of B cells in vitro

To test the conditions under which *F*. *tularensis* LVS is internalized into B cells, the A20 cell line and murine peritoneal cells from naïve as well as immunized BALB/c mice were infected with unopsonized and/or murine fresh serum opsonized or antibody-opsonized *F*. *tularensis* LVS/GFP.

**Fig 1:**
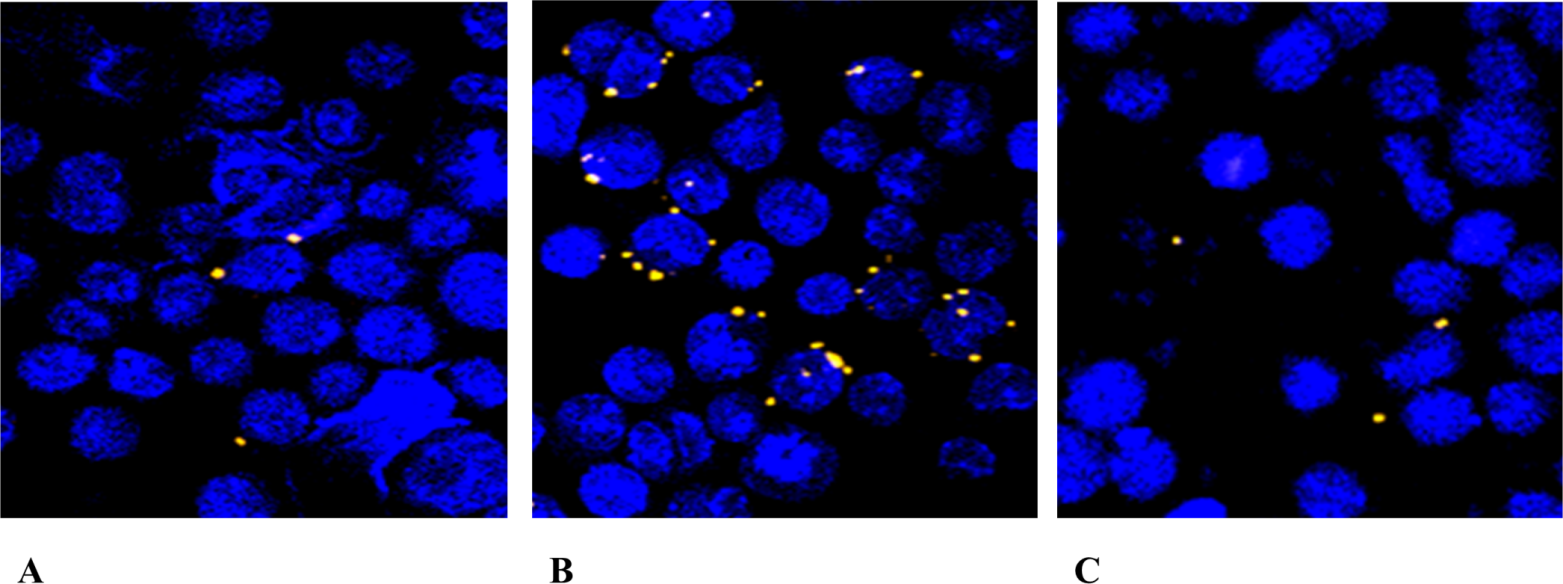
Fluorescent microscopy. The representative picture was chosen to show the difference in the numbers of A20 cells infected with **(A)** unopsonized *F*. *tularensis* LVS/GFP bacteria, **(B)** *F*. *tularensis* LVS/GFP opsonized with murine fresh serum, and **(C)** bacteria opsonized with immune sera. A20 cells in total volume 0.5 mL (1 x 10^6^ cells per well) were infected with *F*. *tularensis* LVS/GFP at MOI 500 for 3 h. The cell nuclei were stained with DAPI. Note: The number of infected cells was counted using flow cytometry.

**Fig 2:**
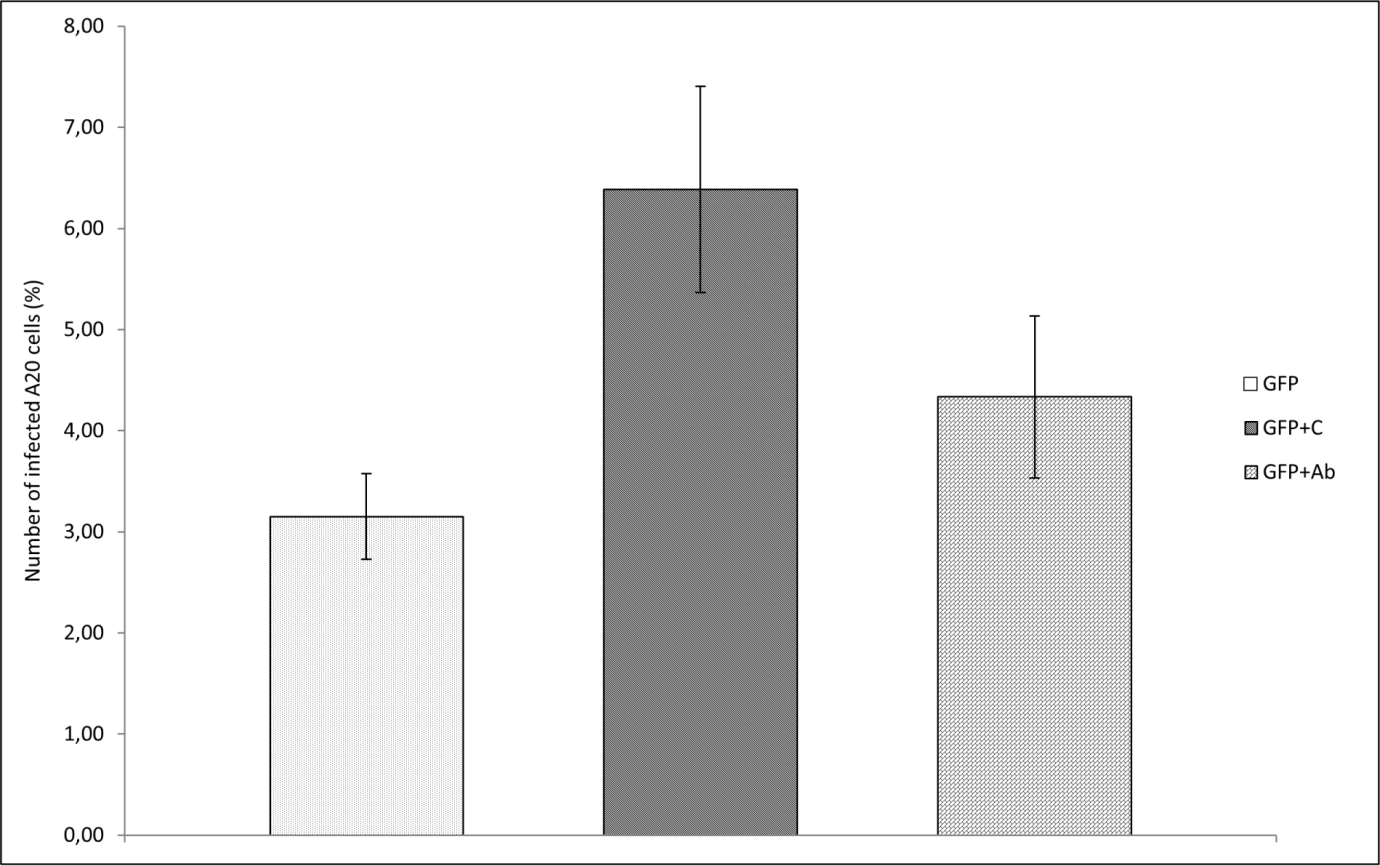
*F*. *tularensis* infecting A20 cells *in vitro*. Cells from the A20 mouse B cell line (5 x 10^5^ per well in total volume 0.5 mL) were infected by *F*. *tularensis* LVS/GFP (GFP) opsonized with fresh uninactivated serum (GFP+C) from naïve mice and bacteria opsonized with heat-inactivated immune sera (GFP+Ab) at MOI 500. The cultures were then incubated at 36.8°C and 5% CO2 atmosphere for 3 h. Proportion of infected cells was determined by flow cytometry. Error bars indicate SD around the means of samples processed in triplicate. Two-tailed *t*-tests were used to test for significant differencess between GFP and GFP+C and GFP+iC and GFP+Ab (*** *P* < 0.001, ** *P* < 0.01). Results shown from one experiment are representative of three independent experiments.

Microscopic inspection of A20 cell cultures infected with *F*. *tularensis* LVS/GFP demonstrated that opsonization of bacteria with murine fresh serum before infection of cultures increased the number of cells with intracellularly localized bacteria (Fig 1). *F*. *tularensis* opsonized with murine fresh serum infected 6.39% of A20 cells while unopsonized *Francisellae* infected only 3.15% of cells, as measured by flow cytometry. Opsonization with antibodies had a less-pronounced effect (Fig 2).

**Fig 3:**
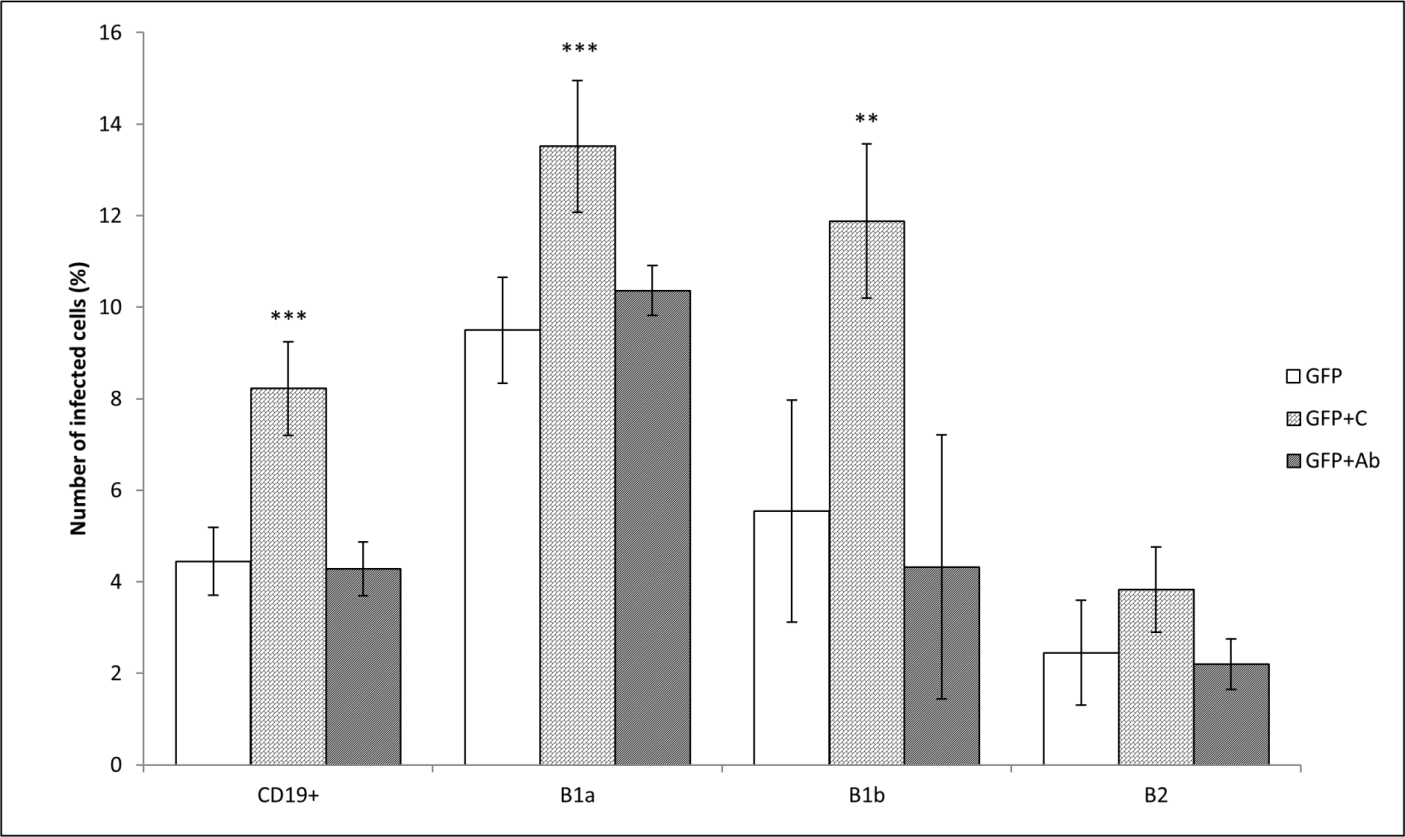
*F*. *tularensis* infecting subsets of B cells *in vitro*. Subsets of B cells were infected for 3 h with unopsonized *F*. *tularensis* LVS/GFP (GFP), *F*. *tularensis* LVS/GFP opsonized with fresh un-inactivated serum (GFP+C) from naïve mice, and bacteria opsonized with heat-inactivated immune sera (GFP+Ab). The proportions of infected CD19^+^ cells from all measured cells and of infected B-1a, B-1b, and B-2 cells from CD19^+^ cells were measured by flow cytometry. Error bars indicate SD around the means of samples processed in triplicate. Two-tailed *t*-test was used to test for significant differences between GFP and GFP+C and GFP+Ab (*** *P* < 0.001, ** *P* < 0.01, * *P* < 0.05). Results shown from one experiment are representative of three independent experiments.

The number of peritoneal CD19^+^ cells infected with unopsonized *F*. *tularensis* fluctuated around 5% (4.44 ± 0.74%). Opsonization of *F*. *tularensis* with fresh un-inactivated serum from naïve mice almost doubled the number of infected cells in culture (8.22 ± 1.02%). Opsonization of bacteria with antibodies–in contrast to opsonization of bacteria with murine fresh serum had no effect (4.28 ± 0.59%). If individual CD19^+^ cell subsets were monitored, then the B-1a cells (CD19^+^CD5^+^CD11b^+^ cells) comprised the dominant subset infected with *Francisellae*. B-1b (CD19^+^CD5^-^CD11b^+^) and B-2 (CD19^+^CD5^-^CD11b^-^) cells were also infected, but at lower frequency than were B-1a cells. Opsonization of bacteria with murine fresh serum or with antibodies in fact copied their effect demonstrated on the entire CD19^+^ cell population with the exception that in B-2 cells the effect of opsonization with fresh murine serum was insignificant (Fig 3). Isotype controls were used for IgG2a (CD19, CD5) and for IgG2b (CD11b). Mean fluorescent intensity for untreated peritoneal control cells was 2.69 ± 0.043, for isotype control IgG2a was 3.50 ± 0.158, for CD19+ (isotype IgG2a) was 454.18 ± 31.641 (*t*-test CD19+ vs IsoC IgG2a *P* = 3.58 x 10^−5^), for CD5+ (isotype IgG2a) was 115.07 ± 6.944 (*t*-test CD5+ vs IsoC IgG2a *P* = 2.16 x 10^−5^), for isotype control IgG2b was 3.54 ± 0.106, and for CD11b+ (IgG2b) was 539.18 ± 56.917 (*t*-test CD11b+ vs IsoC IgG2b P = 1.84 x 10^−4^).

### *Francisella tularensis* strain FSC200 Δ*iglC* and Δ*ftdsbA* deletion mutants failed to enter the A20 cell line

**Fig 4:**
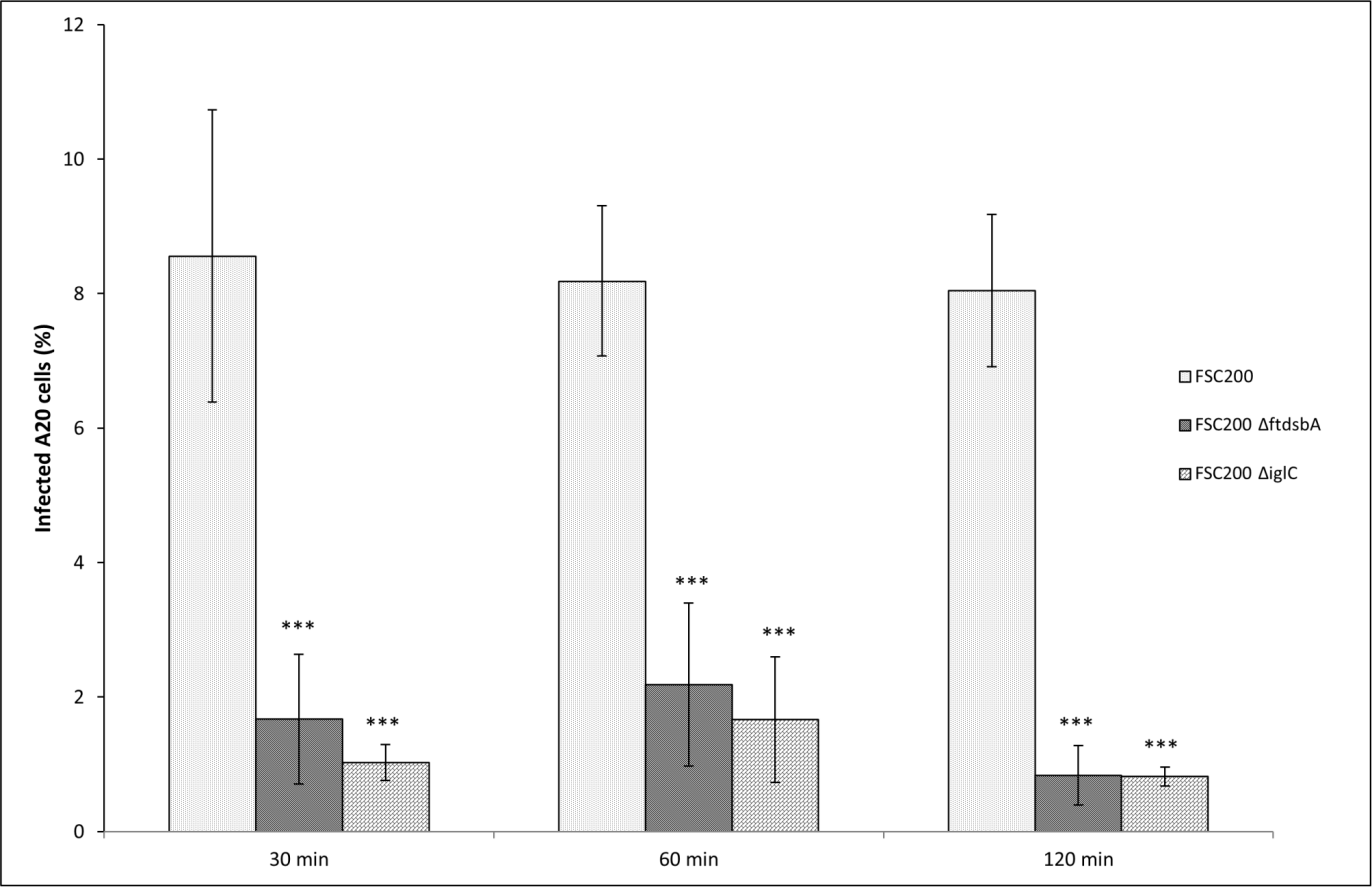
Deletion mutant *F*. *tularensis* strains failed to enter the A20 cells. A20 cells were infected with wild type *F*. *tularensis* FSC200 (FSC200), with deletion mutant *F*. *tularensis* FSC200 Δ*ftdsbA* (FSC200 ΔftdsbA), and with deletion mutant *F*. *tularensis* FSC200 Δ*iglC* (FSC200 ΔiglC), respectively, at MOI 500. The infected cells were determined by florescent microscopy. The cells were stained with DAPI to visualize nuclei and with rabbit anti-*F*. *tularensis sera* and goat anti-rabbit secondary antibody conjugated with Alexa Fluor 488 to visualize *F*. *tularensis*. Error bars indicate SD around the means of samples processed in triplicate. Two-tailed *t*-test was used to test for significant differences between FSC200 and FSC200 ΔftdsbA and FSC200 ΔiglC. (*** *P* < 0.001). Results shown from one experiment are representative of three independent experiments.

Two deletion mutants to *F*. *tularensis* virulence factors IglC and *Francisella* analog to the bacterial DsbA protein (FtdsbA) were used to test the participation of *Francisella* in the internalization process into B cells. The 23 kDa pathogenicity island protein IglC is essential for the survival and proliferation of *F*. *tularensis* in macrophages [44]. The FtdsbA protein is a conserved hypothetical lipoprotein with homology to thiol/disulfide oxidoreductase proteins and is essential for *Francisella* invasivity and intracellular survival [42,45]. Mutants to these significant virulence factors prepared from fully virulent *F*. *tularensis* subsp. *holarctica* strain FCS200 are highly attenuated in virulence for mice in comparison with the original virulent wild strain [42,45]. Both mutants, as they are attenuated in virulence, are also defective in the ability to enter B cells as demonstrated by fluorescent microscopy. During the first 2 h, only around 1% of A20 cells were infected with these mutants (Fig 4). Moreover, the number of bacteria inside infected cells did not exceed 1 bacterium per infected cell. Thus, the genes that control infectivity and virulence of *F*. *tularensis* are important also for the entry of bacteria into B cells.

### Receptor blocking demonstrated the engagement of BCR and CR1/2

To further analyze the entrance gate for *F*. *tularensis* on B cells, we used the blocking antibodies against BCR, CR1/2, CR3, CR4, and FcγR to eliminate their participation in the process of *Francisella* internalization into this cell type. We tested the effect of BCR blocking on the subsets of peritoneal CD19^+^ cells, recognizing that not all B cell subsets utilize, as a response to interaction with bacterium, their unique BCRs for bacterial entry. The effect of other receptor blocking was monitored only on peritoneal CD19^+^ cells as a whole because there are no data indicating that these receptors are not universal in relation to B cell subsets.

**Fig 5:**
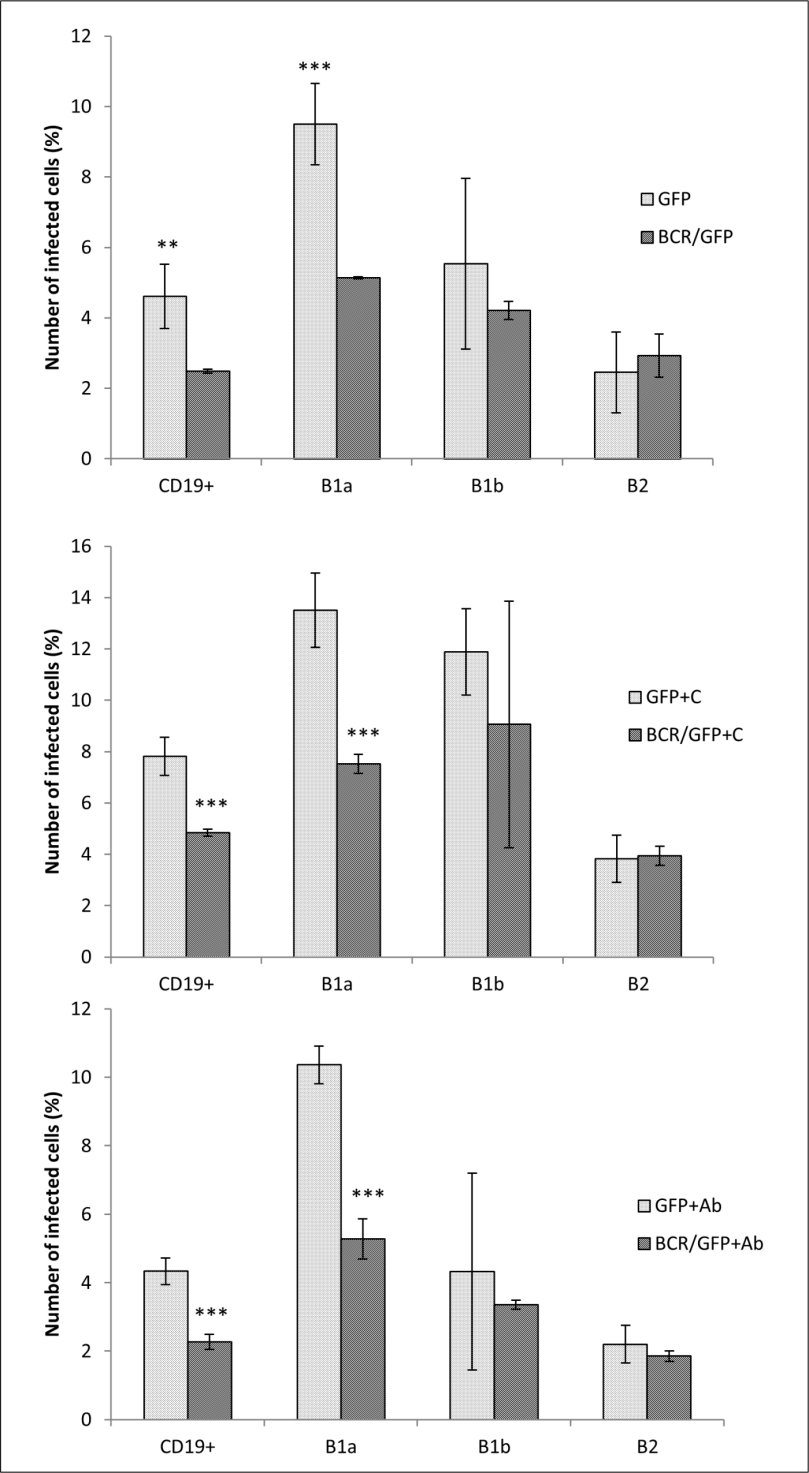
Blocking of BCR receptor. Peritoneal cells were incubated with the blocking antibody anti-IgM (BCR). Thereafter, the cells were infected for 3 h with **(A)** unopsonized *F*. *tularensis* LVS/GFP (GFP), **(B)** *F*. *tularensis* LVS/GFP opsonized with complement (GFP+C), and **(C)** *F*. *tularensis* LVS/GFP opsonized with antibodies (GFP+Ab). Entry into CD19^+^ cells (expressed as percentage of infected CD19^+^ from all CD19^+^ cells) and individual B cell subsets (expressed as percentage of infected B-1a from all B-1a cells, infected B-1b from all B-1b cells, and infected B-2 from all B-2 cells) was detected by flow cytometry. Error bars indicate SD around the means of samples processed in triplicate. Two-tailed *t*-test was used to test for significant differences between untreated cells and cells with blocked BCR (*** *P* < 0.001, ** *P* < 0.01). Results shown from one experiment are representative of three independent experiments.

The effect of BCR blocking on CD19^+^ cells in its entirety and on individual B cell subsets demonstrated that the BCR alone under unopsonized conditions is important only for the entry of *F*. *tularensis* into B-1a cells. The remaining two monitored subsets displayed only insignificant differences in the number of infected cells in unblocked and blocked B cell cultures. Moreover, opsonization with fresh murine serum or antibodies had no effect on the basic difference between B-1a cells on the one hand and B-1b and B2 cells on the other (Fig 5).

**Fig 6:**
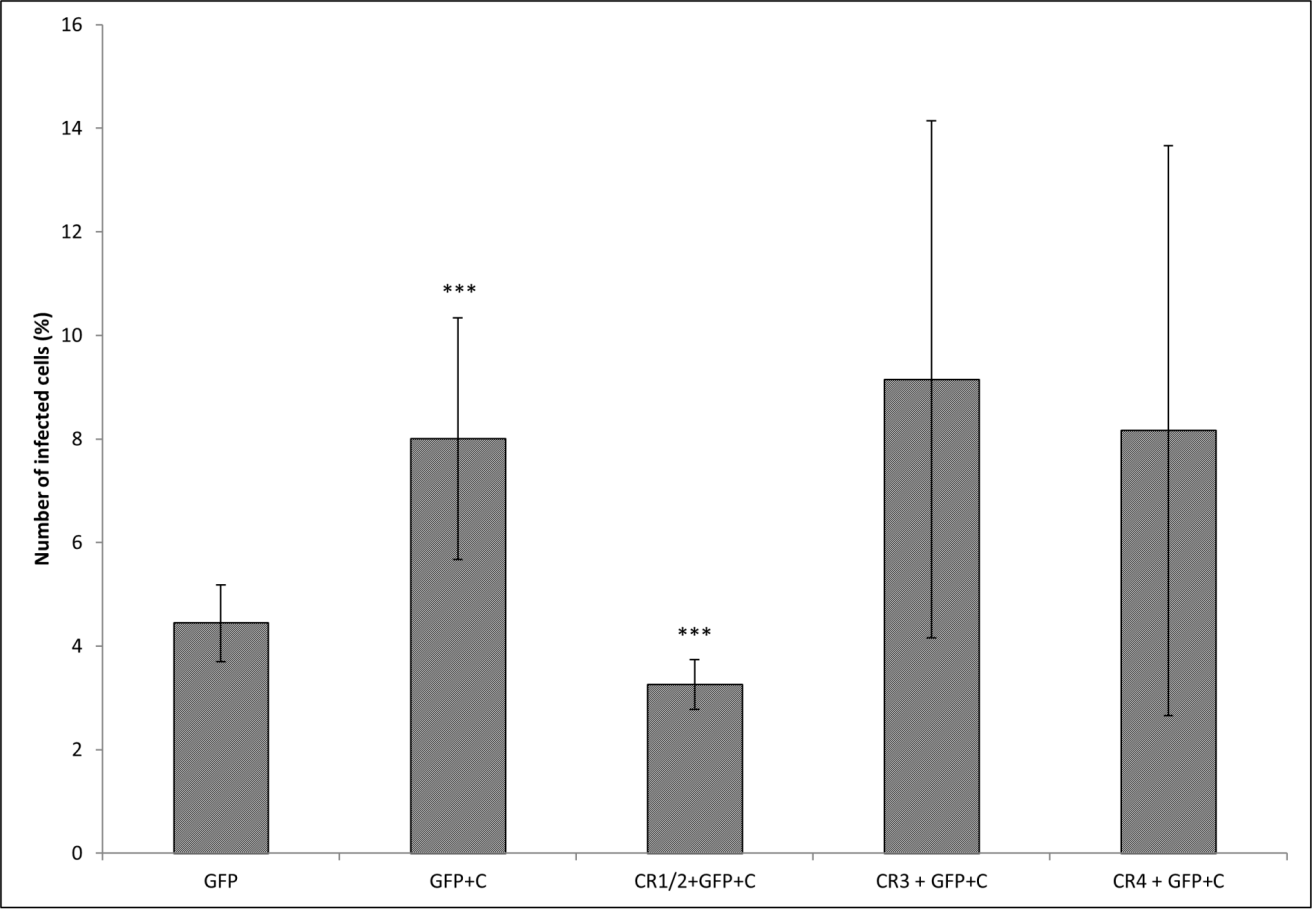
Blocking of CRs. Peritoneal cells were incubated with the antibodies against CD21/CD35 (CR1/2), CD11b (CR3), and CD11c (CR4). After blocking, the cells were infected for 3 h with either *F*. *tularensis* LVS/GFP (GFP) or *F*. *tularensis* LVS/GFP opsonized with complement (GFP+C) and the proportions of infected CD19^+^ cells were detected by flow cytometry. Error bars indicate SD around the means of samples processed in triplicate. Two-tailed *t*-test was used to test for significant differences against GFP. The significance of CR blocking effect was calculated between GFP+C and all groups with blocked CRs (*** *P* < 0.001). Results shown from one experiment are representative of three independent experiments.

The experiments with the effect of CRs blocking on the entry of fresh murine serum opsonized *Francisella* into CD19^+^ cells revealed that of those CRs tested only blocking of CR1/2 significantly reduced the number of infected CD19^+^ cells in *in vitro* cultures. Blocking of CR3 and CR4 had no significant effect (Fig 6).

**Fig 7:**
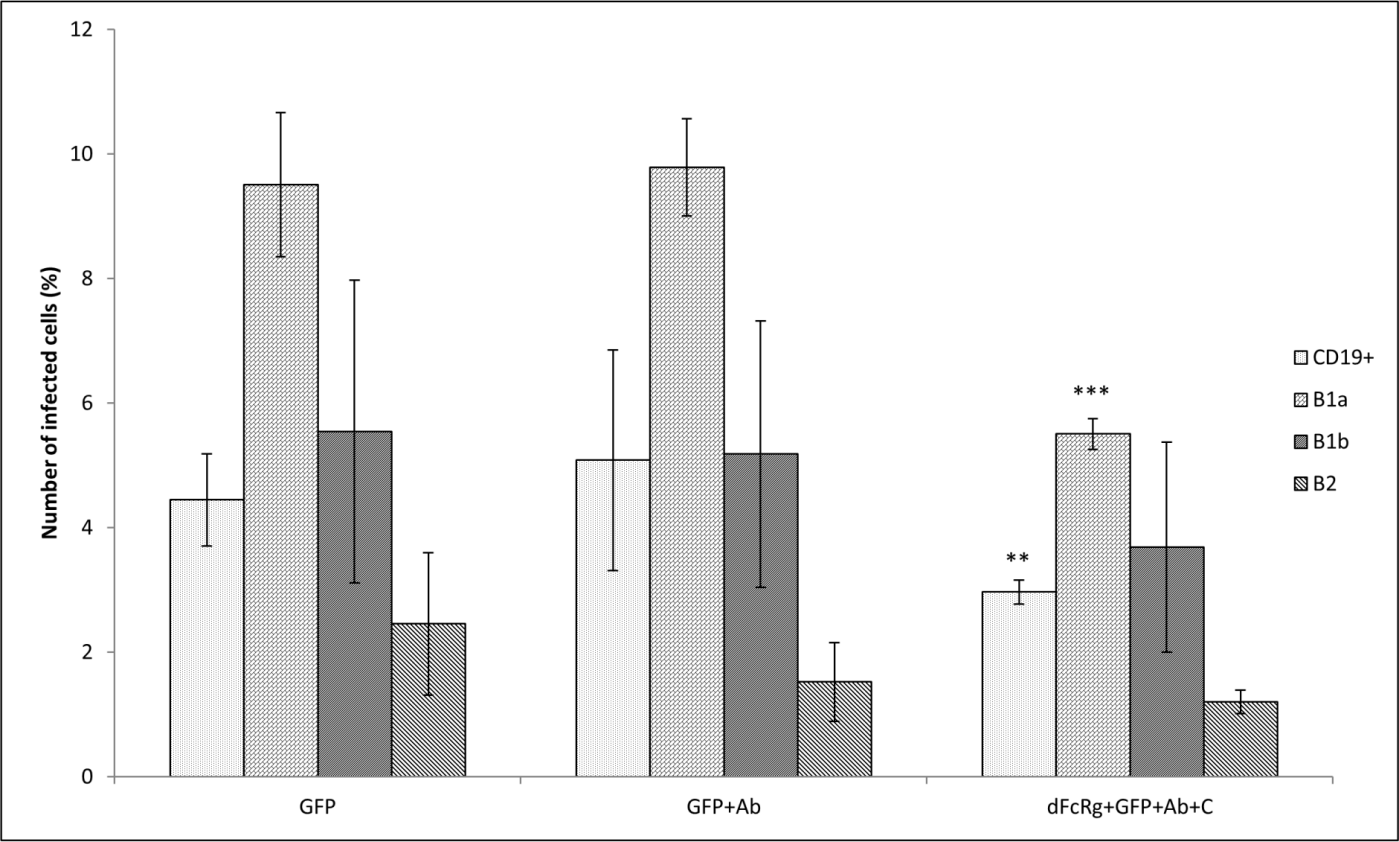
Blocking of FcγR. Peritoneal cells were incubated with the antibody against CD16/32 (dFcRg). Thereafter, cells were infected with *F*. *tularensis* LVS/GFP (GFP), *F*. *tularensis* LVS/GFP opsonized with antibodies (GFP+Ab), and *F*. *tularensis* LVS/GFP opsonized with murine fresh serum and antibodies (dFcRg+GFP+Ab+C) at MOI 500. Entry into all B cells (CD19^+^) and individual B cell subsets was detected 3 h after infection by flow cytometry. Error bars indicate SD around the means of samples processed in triplicate. Two-tailed *t*-test was used to test for significant differences between GFP and GFP+Ab and between GFP+Ab and dFcRg+GFP+Ab+C (*** *P* < 0.001, ** *P* < 0.01). Results shown from one experiment are representative of three independent experiments

The effect of FcγR blocking in experiments with antibody-opsonized *Francisellae* was quite negligible, but repeated experimentation with *Francisellae* opsonized with antibodies and murine fresh serum, representing complement components in their entirety, demonstrated profound decline in the number of infected peritoneal CD19^+^ cells. In subsequent experiments, individual subsets of CD19^+^ cells were monitored. In this case, too, and with the exception of global CD19^+^ cells, only the number of infected B-1a cells was significantly reduced by blocking of FcγR (Fig 7).

### Disturbing lipid rafts

**Fig 8:**
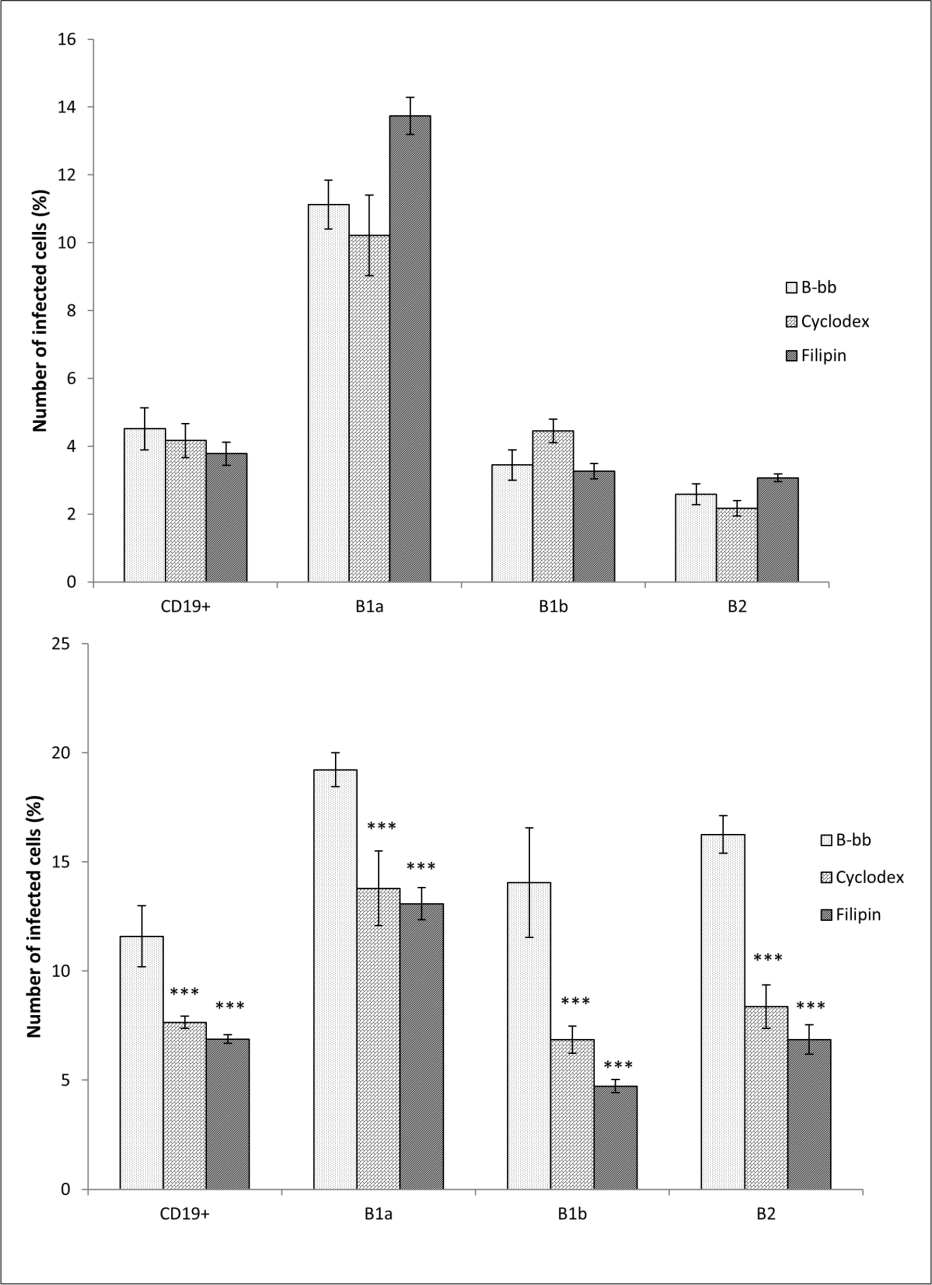
Disturbance of lipid rafts. For disturbing lipid rafts, the cholesterol-binding agent filipin or methyl-beta cyclodextrin (Cyclodex) was used. The peritoneal B cells were pretreated with 10 μg/mL filipin or 10 mM cyclodextrin and consequently infected with **(A)** *F*. *tularensis* LVS/GFP or **(B)** opsonized *F*. *tularensis* LVS/GFP with complement. Entry into all B cells (CD19^+^) and individual B cell subsets was detected by flow cytometry. Error bars indicate SD around the means of samples processed in triplicate. Two-tailed *t*-test was used to test for significant differences between untreated B cells and cyclodextrin- or filipin-treated cells (*** *P* < 0.001). Results shown from one experiment are representative of three independent experiments.

Lipid rafts are well-conserved membrane microdomains that contain high concentrations of cholesterol, sphingolipids, glycosylphosphatidylinositol, GPI-anchored proteins, and dually acylated proteins with kinase characteristics [46]. For disturbing lipid rafts, the cholesterol-binding agent filipin or methyl-beta cyclodextrin (cyclodextrin) was used to evaluate the role of cholesterol-rich membrane domains on internalization of *F*. *tularensis* into peritoneal B cell subsets. Experimental conditions were adjusted so that they could show the effect of opsonization of bacteria with complement, represented here by fresh murine serum, which contributes significantly to infection of B cells by *F*. *tularensis*. Unexpectedly, it was clearly demonstrated that filipin and cyclodextrin inhibited the entry of *F*. *tularensis* into peritoneal CD19^+^ cells only under opsonic conditions. The numbers of cells infected with murine fresh serum opsonized bacteria were reduced by nearly 50% in cultures influenced by cyclodextrin or filipin. Without opsonization, both lipid rafts disturbing agents had only marginal effect on the number of infected CD19^+^ cells. The same insignificant effect was obtained after monitoring individual B cell subsets in cultures infected with nonopsonized bacteria (Fig 8). Considering this together with the results obtained from the experiments with opsonization of bacteria and receptor blocking, it can be concluded that for effective *Francisella* uptake by B cell subsets, the key element from the side of B cells seems to be coordinated construction of a supramolecular receptor structure in lipid rafts. This conclusion was reached based on the assumption that opsonic conditions are much closer to *in vivo* situations than are experimental nonopsonic conditions *in vitro* or *ex vivo*.

### Intracellular trafficking of *Francisella* inside the B cells

**Fig 9:**
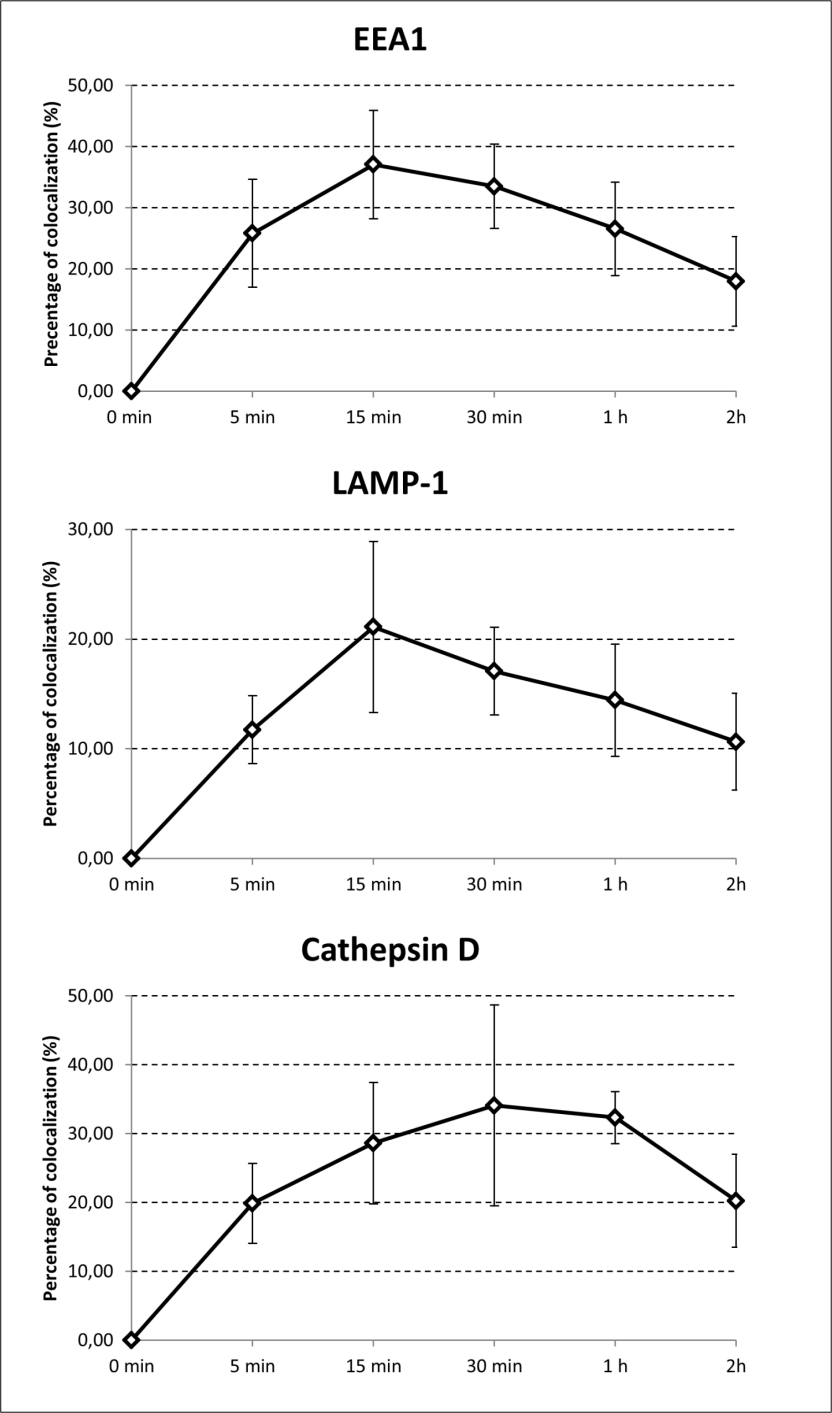
Intracellular trafficking. A20 mouse B cell line (1 x 10^6^ per well in total volume 0.5 mL) was infected with *F*. *tularensis* LVS (MOI 500). Cells were infected for 5, 15 and 30 min, as well as 1 and 2 h. To identify intracellular trafficking, endosomal/lysosomal membrane markers EEA1, LAMP-1, and Cathepsin D were used for determining colocalization of these markers with *F*. *tularensis* LVS by fluorescent microscopy. Error bars indicate SD around the means of samples obtained from three independent experiments.

Once internalized into B cells, *F*. *tularensis*-containing endosomes associate with early endosome antigen 1 (EEA1) followed by the late endosomal/lysosomal membrane marker LAMP-1, and finally they associate with cathepsin D. The association of *F*. *tularensis* LVS-containing endosomes with EEA1 increased from 25.80 ± 8.81% at 5 min to 37.03 ± 8.86% at 15 min, at which time the association with this early endosomal marker terminated. Co-association of *F*. *tularensis* LVS with LAMP-1 increased from 12.74 ± 3.08% at 5 min to 31.25 ± 4.00% at 30 min. The association with cathepsin D peaked 30 min post infection at 34.07 ± 14.58%, and at 2 h post infection the association with cathepsin D still represented 20.20 ± 6.75% (Fig 9). Step-by-step association of *F*. *tularensis*-containing vacuoles with early endosomal marker, late endosomal marker, and finally phago/lysosomal marker indicate that these vacuoles follow the endocytic pathway in its complexity.

It should be noted that when a microscopic study of *F*. *tularensis*–B cell interaction had been carried out, a minority of intracellularly localized *Francisellae* were not localized in membrane-bound compartments [31]. Also in the present case, as can be seen in Fig 9, fewer than 50% of intracellularly localized *Francisellae* colocalized with the endosomal marker used. Intracellular trafficking of *F*. *tularensis* in B cells thus occurs along the endosomal pathway, but without significant multiplication of bacteria, as previously demonstrated [31].

## Discussion

The established functional view of B cells assumes that these cells, which are involved in Ag presentation during initiation of the adaptive phase of immune responses against bacteria, extract antigens from the bacterial surface or, alternatively, from the surface of dendritic cells, or they capture the proteins shed or secreted by bacteria. The dominant feature in this process is immunological synapse formation [47,48]. This standard view was based on the conviction that B cells, as non-phagocytic cells, lack the ability to internalize particulate antigens. Recently, however, there are data showing that bacterial pathogens, including intracellular bacteria, are internalized (or actively enter) into murine as well as human B cells and B cell lines. *Salmonellae* survive inside B cell lines, mouse spleen B cells, B cell precursors, as well as plasma cells in murine bone marrow [38,49]. *Listeria monocytogenes* can infect virtually all cell types, including primary or transformed B cells [50]. B lymphocytes, pre-B cells, and B blasts bind, ingest, and permit multiplication of such pathogens as *Chlamydia trachomatis* [36] and *Mycobacterium tuberculosis* [51] or opsonized *Brucella abortus* [37]. *F*. *tularensis*, another intracellular pathogen, also infects murine primary B cells and murine and human B cell lines, as we have shown previously [31]. The entry of intracellular bacteria into B cells or B cell precursors thus seems to be a common phenomenon, albeit one restricted to only a limited part of the B cell population. Typically, only about 5% to 10% of B cells in culture have been documented as infected.

In our experimental setup, the first contact of *Francisella* with the B cell was characterized by the creation of a tight junction between bacterial and eukaryotic cell membranes [31]. The area of *Francisella* and B cell mutual contact is microscopically reminiscent of the immunological synapse formation [47]. Subsequently, a significant number of CD19^+^ cells engulf *Francisellae*. The increased proportion of infected B cells can be detected under opsonic conditions when fresh murine serum from naïve mice has been used in completing the cultivation medium (see S1 Table.). Opsonization with heat-inactivated immune serum, unlike fresh murine serum opsonization, had no significant effect on *Francisella* engulfment by B cells. *Francisellae* localized in intracellular space of B cells did not proliferate but did remain alive. We were able to cultivate bacterial colonies after seeding separated CD19^+^ peritoneal cells onto McLeod plates 24 h after infection (see S1 Fig).

Blocking experiments documented BCR and CR1/2 engagement in the process of recognizing and engulfing *Francisella* into B cells under *ex vivo* conditions. The CR3 and CR4 are substantially not involved in these processes. Thus, the receptor profile engaged in the internalization of *Francisellae* demonstrated here, together with the activation markers and co-receptors CD80 and CD86 expression demonstrated in the previous publication [31], can ensure, inter alia, optimal immune response of CD19^+^ cells.

According to the data presented here, the BCR utilizes CR1/2 not only for expressing optimal adaptive humoral immune response (for review, see [52]) but also for recognition and consequent internalization of bacteria into B cells, which is precisely in accordance with the concept of dual antigen recognition by B cells [52–54]. These events are occurring at the periphery, thus suggesting that peripheral B cells are among the active cellular components participating in innate immune responses against bacteria.

To initiate the process of *F*. *tularensis* internalization into CD19^+^ cells under opsonizing conditions, the B cell membrane lipid bilayer must be fluid and contain cholesterol because filipin or cyclodextrin significantly reduces the number of intracellularly localized *Francisellae*. Filipin may form large planar aggregates between the two layers of the membrane. It may be absorbed at the membrane surface, may be located at the upper layer of the membrane, and can form the filipin–sterol complexes within the membrane bilayer. Cyclodextrin enhances cholesterol solubility and thereby causes the instability of cholesterol-enriched lipid rafts [55]. Bacteria alone, as multivalent corpuscular antigens, can cluster the BCR and in the presence of complement can span both BCRs and CRs. Moreover, it seems likely that bacteria crosslinking BCRs and CRs orient these into a common membrane domain. Also in this respect, therefore, the process of bacteria internalization into B cells corresponds to the process involved in BCR activation, as well as in antigen capture and processing. In the latter case, BCR signaling requires BCR clustering where the tetraspanin microdomains and/or lipid rafts are involved [56,57]. Interestingly, when the complement is not involved in the primary mutual interaction of the bacterium with the B cell, the BCR of B cells–minimally of B1a cells–probably specific to bacterial antigens alone, seems sufficient to achieve the signals for internalization of surface-bound bacteria. In our case, during the B cell–*Francisella* primary interaction, the term “specific to bacterial antigen” means not only B cells specific to unique *Francisella* antigens (i.e., so-called hypothetical proteins) but also B cells specific to pan-bacterial markers including pathogen-associated molecular paterns (PAMPs) carried by *Francisella*. In our experience, the sera from naïve SPF mice reacted in western blots with several *F*. *tularensis* proteins that are not unique proteins of *Francisellae*.

Taken together, the initial events seem to constitute a special example of a common antigenic-recognition phenomenon during internalization of intracellular bacteria into B cells. Whether or not the *Francisellae* actively participate in the process of their internalization into this cell type is still unknown. Nevertheless, *F*. *tularensis* strain FSC200 Δ*iglC* and Δ*ftdsbA* deletion mutants are defective in the ability to enter B cells. The *iglC* gene is coded in a highly conserved *Francisella* pathogenicity island (FPI), a region of 16 to 19 protein-coding genes from which a subset of genes share limited homology with core structural components of a Type VI secretion system (T6SS) and which are needed for delivery of virulence factors into host cells [58,59]. The gene for a conserved hypothetical lipoprotein with homology to thiol/disulfide oxidoreductase proteins, a DsbA homologue, designated also as FipA [60], is likely responsible for proper folding of substrates having virulence factor character [42,61,62]. It thus seems likely that if the FPI codes the genes for functional T6SS, then secretion of virulence factors that are substrates of Fip A will be, at minimum, indispensable for successful entry of *F*. *tularensis* into B cells. Whether the role of T6SS and/or FipA consists in transferring the ligands of host receptors in proper conformation to bacterial surface or in modulation of host cell internalization mechanisms remains to be elucidated. Moreover, killed, formaldehyde vapor-treated, *F*. *tularensis* FSC200 does not enter B cells [31].

*Francisella* infect only a small, but still significant number of B cells. What factors define the ability to be infected is unclear. When the BCR is engaged, the possibility exists that the criterion for infection may be the specificity of this BCR for the given antigen. Selective infection of antigen-specific B cells by *Salmonella* already has been demonstrated [41]. The existence of B cell subsets can be another reason for the uneven ability to engulf bacteria. Peritoneal cavity murine B-1 cells can differentiate into a mononuclear phagocyte *in vitro* and acquire the ability to phagocytize large particles [63]. In the *ex vivo* experimental system presented here, all tested subsets of peritoneal B cells, meaning B-1a, B-1b, and B-2 cells, were infected with unequal frequency. This inequality in ability to be infected became more distinct when bacteria were opsonized with murine fresh serum. In this case, B-1b and B-2 cells were more frequently infected than were B-1a cells. Interestingly, only peritoneal B-1a cells were identified to engulf, or to be infected by, *F*. *tularensis* after *in vivo* intraperitoneal infection [32]. Thus, the BCRs of B-1a cells alone seem to be sufficient for generation of signals enabling the engulfment of bacteria, and the co-signaling from CR1/2 has only a minimal impact on the functional profile of B-1a cells that interact with bacterium. In contrast to B-1a cells, B-1b and B-2 cells seem to need the cooperation of their BCRs with CR1/2 to generate signals that initiate engulfment of *F*. *tularensis*. Recent studies have shown that B cell subsets of mammalian B cells in general have phagocytic activity. Nevertheless, only a proportion of cells of individual B cell subsets show phagocytic activity to stimulation with particles. Research has shown that 14–17% of B-1a cells, 8.6–11.4% of B-1b cells, and less than 1.5% of B-2 B cells in the peritoneal cavity demonstrated phagocytic activity both *in vitro* and *in vivo* [64,65]. Whether or not the internalization of *F*. *tularensis* into B-1a cells, on the one hand, and internalization into B-1b or B-2 cells, on the other, is carried out under the same signaling scenario during *in vivo* and *in vitro* studies is recently unclear. On the one hand, this may be macropinocytosis, as has been demonstrated for *Mycobacteriae* and *Salmonellae* [39,51]. On the other hand, it may be classical opsonophagocytosis as a dominant mechanism for internalization of particles.

In summary, we can conclude that *F*. *tularensis* actively induces entry into B cells. The results presented here suggest that BCRs alone at the B-1a subset can ensure the internalization process. The BCRs on B-1b and B-2 cells need co-signaling from the co-receptor containing the CR1/2 to initiate *F*. *tularensis* engulfment. In this case, the fluidity of the surface cell membrane is a prerequisite for bacteria internalization.

## Supporting Information

S1 Fig Relative infectivity of *F*. *tularensis* LVS opsonized with fresh murine sera (C) and inactivated fresh murine sera (iC). The volume of 500 μL of fresh murine sera (C) or 500 μL of murine heat-inactivated fresh sera (iC) as a control were added to 4 x 10^9^ bacteria, incubated at 36.8°C for 1 h, washed twice with pre-warmed PBS, resuspended in 1 mL saline, and used for the experiments. To opsonized bacteria the sera were added to 1 mL of bacterial suspension. A20 cells (5 x 10^5^ per well) were co-cultivated with unopsonized, opsonized with C, and iC resp. *F*. *tularensis* LVS/GFP in total volume of 0.5 mL per well at MOI 500. Control A20 cells were cultivated without infection. After 3 h incubation cultures were washed using PBS at 36.8°C and 5% CO_2_ atmosphere. The proportion of infected cells with *F*. *tularensis* LVS/GFP was examined using flow cytometry. Error bars indicate SD around the means of samples processed in triplicate. Relative infectivity was calculated as a ratio among A20 cells infected with *F*. *tularensis* LVS/GFP and A20 cells infected with opsonized bacteria. Proportional number of infected A20 cells with GFP was laid to be one. Two-tailed *t*-test was used to find significant difference between GFP and GFP+C and, GFP+iC (* *P* < 0.05). Results shown from one experiment are representative of three independent experiments. Note: Heat-inactivated sera were prepared by heating sera in water bath (56°C) at the volume of 0.5 mL for 30 min.
(PDF)

S1 Table Viability of bacteria inside the B cells.BALB/c mice were infected with *F*. *tularensis* LVS/GFP. After 24 h peritoneal cells were collected, resuspended in DMEM cultivation medium supplemented with 2% fetal bovine serum and then incubated with the antibody CD19-Alexa Fluor 647. Peritoneal CD19^+^ cells were sorted using BD FACSAria II Cell Sorter. Sorted CD19^+^ cells were washed using PBS and lysed by 0.1% sodium deoxycholate after washing. Actual numbers of bacteria were determined by serial dilutions (10^0^ and 10^−2^) and the number of CFU was calculated. ^1^ Number of CD19^+^ cells seeded onto McLeod plates in volume 50 μL and cultivated at 36.8°C. ^2^ The number of CFU was determined after 48–72 h of cultivation.
(PDF)

## References

[1] Detilleux PG, Deyoe BL, Cheville NF. Penetration and intracellular growth of *Brucella abortus* in nonphagocytic cells in vitro. Infect Immun. 1990;58: 2320–2328. 2114362 10.1128/iai.58.7.2320-2328.1990 PMC258815

[2] Pizarro-Cerdá J, Méresse S, Parton RG, van der Goot G, Sola-Landa A, Lopez-Goñi I, et al *Brucella abortus* transits through the autophagic pathway and replicates in the endoplasmic reticulum of nonprofessional phagocytes. Infect Immun. 1998;66: 5711–5724. 9826346 10.1128/iai.66.12.5711-5724.1998 PMC108722

[3] Pizarro-Cerdá J, Moreno E, Gorvel JP. Invasion and intracellular trafficking of *Brucella abortus* in nonphagocytic cells. Microbes Infect Inst Pasteur. 2000;2: 829–835. 10.1016/s1286-4579(00)90368-x 10955964

[4] Vitry M-A, Hanot Mambres D, Deghelt M, Hack K, Machelart A, Lhomme F, et al *Brucella melitensis* Invades Murine Erythrocytes during Infection. Infect Immun. 2014;82: 3927–3938. 10.1128/IAI.01779-14 25001604 PMC4187840

[5] Velge P, Wiedemann A, Rosselin M, Abed N, Boumart Z, Chaussé AM, et al Multiplicity of *Salmonella* entry mechanisms, a new paradigm for *Salmonella* pathogenesis. MicrobiologyOpen. 2012;1: 243–258. 10.1002/mbo3.28 23170225 PMC3496970

[6] Veiga E, Cossart P. The role of clathrin-dependent endocytosis in bacterial internalization. Trends Cell Biol. 2006;16: 499–504. 10.1016/j.tcb.2006.08.005 16962776 PMC7126422

[7] Bonazzi M, Lecuit M, Cossart P. *Listeria monocytogenes* internalin and E-cadherin: from structure to pathogenesis. Cell Microbiol. 2009;11: 693–702. 10.1111/j.1462-5822.2009.01293.x 19191787

[8] Mostowy S, Cossart P. Cytoskeleton rearrangements during *Listeria* infection: clathrin and septins as new players in the game. Cell Motil Cytoskeleton. 2009;66: 816–823. 10.1002/cm.20353 19296488

[9] Pizarro-Cerdá J, Kühbacher A, Cossart P. Entry of *Listeria monocytogenes* in mammalian epithelial cells: an updated view. Cold Spring Harb Perspect Med. 2012;2 10.1101/cshperspect.a010009 PMC3543101 23125201

[10] Santic M, Molmeret M, Klose KE, Abu Kwaik Y. *Francisella tularensis* travels a novel, twisted road within macrophages. Trends Microbiol. 2006;14: 37–44. 10.1016/j.tim.2005.11.008 16356719

[11] Santic M, Al-Khodor S, Abu Kwaik Y. Cell biology and molecular ecology of *Francisella tularensis* . Cell Microbiol. 2010;12: 129–139. 10.1111/j.1462-5822.2009.01400.x 19863554

[12] Moreau GB, Mann BJ. Adherence and uptake of *Francisella* into host cells. Virulence. 2013;4: 826–832. 10.4161/viru.25629 23921460 PMC3925714

[13] Forestal CA, Malik M, Catlett SV, Savitt AG, Benach JL, Sellati TJ, et al *Francisella tularensis* has a significant extracellular phase in infected mice. J Infect Dis. 2007;196: 134–137. 10.1086/518611 17538893

[14] Bolger CE, Forestal CA, Italo JK, Benach JL, Furie MB. The live vaccine strain of *Francisella tularensis* replicates in human and murine macrophages but induces only the human cells to secrete proinflammatory cytokines. J Leukoc Biol. 2005;77: 893–897. 10.1189/jlb.1104637 15758077

[15] Sjöstedt A. Intracellular survival mechanisms of *Francisella tularensis*, a stealth pathogen. Microbes Infect Inst Pasteur. 2006;8: 561–567. 10.1016/j.micinf.2005.08.001 16239121

[16] Clemens DL, Horwitz MA. Uptake and intracellular fate of *Francisella tularensis* in human macrophages. Ann N Y Acad Sci. 2007;1105: 160–186. 10.1196/annals.1409.001 17435118

[17] Ramond E, Gesbert G, Barel M, Charbit A. Proteins involved in *Francisella tularensis* survival and replication inside macrophages. Future Microbiol. 2012;7: 1255–1268. 10.2217/fmb.12.103 23075445

[18] Hall JD, Woolard MD, Gunn BM, Craven RR, Taft-Benz S, Frelinger JA, et al Infected-host-cell repertoire and cellular response in the lung following inhalation of *Francisella tularensis* Schu S4, LVS, or U112. Infect Immun. 2008;76: 5843–5852. 10.1128/IAI.01176-08 18852251 PMC2583552

[19] Law HT, Lin AE-J, Kim Y, Quach B, Nano FE, Guttman JA. *Francisella tularensis* uses cholesterol and clathrin-based endocytic mechanisms to invade hepatocytes. Sci Rep. 2011;1: 192 10.1038/srep00192 22355707 PMC3240981

[20] Meibom KL, Barel M, Charbit A. Loops and networks in control of *Francisella tularensis* virulence. Future Microbiol. 2009;4: 713–729. 10.2217/fmb.09.37 19659427

[21] Horzempa J, O’Dee DM, Stolz DB, Franks JM, Clay D, Nau GJ. Invasion of erythrocytes by *Francisella tularensis* . J Infect Dis. 2011;204: 51–59. 10.1093/infdis/jir221 21628658 PMC3105038

[22] Malik M, Bakshi CS, Sahay B, Shah A, Lotz SA, Sellati TJ. Toll-like receptor 2 is required for control of pulmonary infection with *Francisella tularensis* . Infect Immun. 2006;74: 3657–3662. 10.1128/IAI.02030-05 16714598 PMC1479238

[23] Abplanalp AL, Morris IR, Parida BK, Teale JM, Berton MT. TLR-dependent control of *Francisella tularensis* infection and host inflammatory responses. PloS One. 2009;4: e7920 10.1371/journal.pone.0007920 19936231 PMC2775407

[24] Chakraborty S, Monfett M, Maier TM, Benach JL, Frank DW, Thanassi DG. Type IV pili in *Francisella tularensis*: roles of pilF and pilT in fiber assembly, host cell adherence, and virulence. Infect Immun. 2008;76: 2852–2861. 10.1128/IAI.01726-07 18426883 PMC2446743

[25] Melillo A, Sledjeski DD, Lipski S, Wooten RM, Basrur V, Lafontaine ER. Identification of a *Francisella tularensis* LVS outer membrane protein that confers adherence to A549 human lung cells. FEMS Microbiol Lett. 2006;263: 102–108. 10.1111/j.1574-6968.2006.00413.x 16958857

[26] Barel M, Hovanessian AG, Meibom K, Briand J-P, Dupuis M, Charbit A. A novel receptor—ligand pathway for entry of *Francisella tularensis* in monocyte-like THP-1 cells: interaction between surface nucleolin and bacterial elongation factor Tu. BMC Microbiol. 2008;8: 145 10.1186/1471-2180-8-145 18789156 PMC2551611

[27] Lindemann SR, McLendon MK, Apicella MA, Jones BD. An In Vitro Model System Used To Study Adherence and Invasion of *Francisella tularensis* Live Vaccine Strain in Nonphagocytic Cells. Infect Immun. 2007;75: 3178–3182. 10.1128/IAI.01811-06 17339345 PMC1932879

[28] Craven RR, Hall JD, Fuller JR, Taft-Benz S, Kawula TH. *Francisella tularensis* invasion of lung epithelial cells. Infect Immun. 2008;76: 2833–2842. 10.1128/IAI.00043-08 18426871 PMC2446690

[29] Schulert GS, Allen L-AH. Differential infection of mononuclear phagocytes by *Francisella tularensis*: role of the macrophage mannose receptor. J Leukoc Biol. 2006;80: 563–571. 10.1189/jlb.0306219 16816147 PMC1865506

[30] Geier H, Celli J. Phagocytic receptors dictate phagosomal escape and intracellular proliferation of *Francisella tularensis* . Infect Immun. 2011;79: 2204–2214. 10.1128/IAI.01382-10 21422184 PMC3125850

[31] Krocova Z, Härtlova A, Souckova D, Zivna L, Kroca M, Rudolf E, et al Interaction of B cells with intracellular pathogen *Francisella tularensis* . Microb Pathog. 2008;45: 79–85. 10.1016/j.micpath.2008.01.010 18524531

[32] Plzakova L, Kubelkova K, Krocova Z, Zarybnicka L, Sinkorova Z, Macela A. B cell subsets are activated and produce cytokines during early phases of *Francisella tularensis* LVS infection. Microb Pathog. 2014;75C: 49–58. 10.1016/j.micpath.2014.08.009 25200734

[33] Bratescu A, Mayer EP, Teodorescu M. Binding of bacteria from the genus *Brucella* to human B lymphocytes. Infect Immun. 1981;31: 816–821. 6783546 10.1128/iai.31.2.816-821.1981 PMC351382

[34] Teodorescu M, Mayer EP. Binding of bacteria to lymphocyte subpopulations. Adv Immunol. 1982;33: 307–351. 6215839 10.1016/s0065-2776(08)60838-x

[35] Lombardi G, Del Gallo F, Vismara D, Piccolella E, Colizzi V. Immunology of tuberculosis: new directions in research. Ric Clin Lab. 1987;17: 1–15. 3109004 10.1007/BF02909383

[36] Bard JA, Levitt D. Binding, ingestion, and multiplication of *Chlamydia trachomatis* (L2 serovar) in human leukocyte cell lines. Infect Immun. 1985;50: 935–937. 4066039 10.1128/iai.50.3.935-937.1985 PMC261174

[37] Goenka R, Guirnalda PD, Black SJ, Baldwin CL. B Lymphocytes provide an infection niche for intracellular bacterium *Brucella abortus* . J Infect Dis. 2012;206: 91–98. 10.1093/infdis/jis310 22561364 PMC3415929

[38] Verjans GM, Ringrose JH, van Alphen L, Feltkamp TE, Kusters JG. Entrance and survival of *Salmonella typhimurium* and *Yersinia enterocolitica* within human B- and T-cell lines. Infect Immun. 1994;62: 2229–2235. 7514574 10.1128/iai.62.6.2229-2235.1994 PMC186502

[39] Rosales-Reyes R, Pérez-López A, Sánchez-Gómez C, Hernández-Mote RR, Castro-Eguiluz D, Ortiz-Navarrete V, et al Salmonella infects B cells by macropinocytosis and formation of spacious phagosomes but does not induce pyroptosis in favor of its survival. Microb Pathog. 2012;52: 367–374. 10.1016/j.micpath.2012.03.007 22475626

[40] Souwer Y, Griekspoor A, Jorritsma T, de Wit J, Janssen H, Neefjes J, et al B cell receptor-mediated internalization of *Salmonella*: a novel pathway for autonomous B cell activation and antibody production. J Immunol Baltim Md 1950. 2009;182: 7473–7481. 10.4049/jimmunol.0802831 19494270

[41] Souwer Y, Griekspoor A, de Wit J, Martinoli C, Zagato E, Janssen H, et al Selective infection of antigen-specific B lymphocytes by *Salmonella* mediates bacterial survival and systemic spreading of infection. PloS One. 2012;7: e50667 10.1371/journal.pone.0050667 23209805 PMC3510171

[42] Straskova A, Pavkova I, Link M, Forslund A-L, Kuoppa K, Noppa L, et al Proteome analysis of an attenuated *Francisella tularensis* dsbA mutant: identification of potential DsbA substrate proteins. J Proteome Res. 2009;8: 5336–5346. 10.1021/pr900570b 19799467

[43] Golovliov I, Sjöstedt A, Mokrievich A, Pavlov V. A method for allelic replacement in *Francisella tularensis* . FEMS Microbiol Lett. 2003;222: 273–280. 12770718 10.1016/S0378-1097(03)00313-6

[44] Santic M, Molmeret M, Klose KE, Jones S, Kwaik YA. The *Francisella tularensis* pathogenicity island protein IglC and its regulator MglA are essential for modulating phagosome biogenesis and subsequent bacterial escape into the cytoplasm. Cell Microbiol. 2005;7: 969–979. 10.1111/j.1462-5822.2005.00526.x 15953029

[45] Qin A, Scott DW, Thompson JA, Mann BJ. Identification of an essential *Francisella tularensis* subsp. *tularensis* virulence factor. Infect Immun. 2009;77: 152–161. 10.1128/IAI.01113-08 18981253 PMC2612291

[46] Pike LJ. The challenge of lipid rafts. J Lipid Res. 2009;50: S323–S328. 10.1194/jlr.R800040-JLR200 18955730 PMC2674732

[47] Batista FD, Iber D, Neuberger MS. B cells acquire antigen from target cells after synapse formation. Nature. 2001;411: 489–494. 10.1038/35078099 11373683

[48] Batista FD, Harwood NE. The who, how and where of antigen presentation to B cells. Nat Rev Immunol. 2009;9: 15–27. 10.1038/nri2454 19079135

[49] Castro-Eguiluz D, Pelayo R, Rosales-Garcia V, Rosales-Reyes R, Alpuche-Aranda C, Ortiz-Navarrete V. B cell precursors are targets for *Salmonella* infection. Microb Pathog. 2009;47: 52–56. 10.1016/j.micpath.2009.04.005 19383536

[50] Menon A, Shroyer ML, Wampler JL, Chawan CB, Bhunia AK. In vitro study of *Listeria monocytogenes* infection to murine primary and human transformed B cells. Comp Immunol Microbiol Infect Dis. 2003;26: 157–174. 12581746 10.1016/s0147-9571(02)00039-5

[51] García-Pérez BE, De la Cruz-López JJ, Castañeda-Sánchez JI, Muñóz-Duarte AR, Hernández-Pérez AD, Villegas-Castrejón H, et al Macropinocytosis is responsible for the uptake of pathogenic and non-pathogenic mycobacteria by B lymphocytes (Raji cells). BMC Microbiol. 2012;12: 246 10.1186/1471-2180-12-246 23113903 PMC3559283

[52] Carroll MC, Isenman DE. Regulation of humoral immunity by complement. Immunity. 2012;37: 199–207. 10.1016/j.immuni.2012.08.002 22921118 PMC5784422

[53] Van Noesel CJ, Lankester AC, van Lier RA. Dual antigen recognition by B cells. Immunol Today. 1993;14: 8–11. 7680204 10.1016/0167-5699(93)90316-d

[54] Heyman B. A third way to induce crosslinking of membrane immunoglobulin and CR2? Immunol Today. 1993;14: 515 10.1016/0167-5699(93)90269-Q 8123146

[55] Gimpl G, Gehring-Burger K. Cholesterol Reporter Molecules. Biosci Rep. 2007;27: 335–358. 17668316 10.1007/s10540-007-9060-1

[56] Schmidt C, Kim D, Ippolito GC, Naqvi HR, Probst L, Mathur S, et al Signalling of the BCR is regulated by a lipid rafts-localised transcription factor, Bright. EMBO J. 2009;28: 711–724. 10.1038/emboj.2009.20 19214191 PMC2666038

[57] Zuidscherwoude M, de Winde CM, Cambi A, van Spriel AB. Microdomains in the membrane landscape shape antigen-presenting cell function. J Leukoc Biol. 2014;95: 251–263. 10.1189/jlb.0813440 24168856

[58] Bröms JE, Meyer L, Sun K, Lavander M, Sjöstedt A. Unique substrates secreted by the type VI secretion system of *Francisella tularensis* during intramacrophage infection. PloS One. 2012;7: e50473 10.1371/journal.pone.0050473 23185631 PMC3502320

[59] Law HT, Sriram A, Fevang C, Nix EB, Nano FE, Guttman JA. IglC and PdpA are important for promoting *Francisella* invasion and intracellular growth in epithelial cells. PloS One. 2014;9: e104881 10.1371/journal.pone.0104881 25115488 PMC4130613

[60] Qin A, Scott DW, Rabideau MM, Moore EA, Mann BJ. Requirement of the CXXC motif of novel *Francisella* infectivity potentiator protein B FipB, and FipA in virulence of *F*. *tularensis* subsp. *tularensis* . PloS One. 2011;6: e24611 10.1371/journal.pone.0024611 21931773 PMC3169626

[61] Qin A, Zhang Y, Clark ME, Rabideau MM, Millan Barea LR, Mann BJ. FipB, an essential virulence factor of *Francisella tularensis* subspecies tularensis, has dual roles in disulfide bond formation. J Bacteriol. 2014; 10.1128/JB.01359-13 PMC4187702 25092026

[62] Ren G, Champion MM, Huntley JF. Identification of disulfide bond isomerase substrates reveals bacterial virulence factors. Mol Microbiol. 2014;94: 926–944. 10.1111/mmi.12808 25257164 PMC4227921

[63] Almeida SR, Aroeira LS, Frymuller E, Dias MA, Bogsan CS, Lopes JD, et al Mouse B-1 cell-derived mononuclear phagocyte, a novel cellular component of acute non-specific inflammatory exudate. Int Immunol. 2001;13: 1193–1201. 11526100 10.1093/intimm/13.9.1193

[64] Parra D, Rieger AM, Li J, Zhang Y-A, Randall LM, Hunter CA, et al Pivotal advance: peritoneal cavity B-1 B cells have phagocytic and microbicidal capacities and present phagocytosed antigen to CD4+ T cells. J Leukoc Biol. 2012;91: 525–536. 10.1189/jlb.0711372 22058420 PMC3317272

[65] Sunyer JO. Evolutionary and functional relationships of B cells from fish and mammals: insights into their novel roles in phagocytosis and presentation of particulate antigen. Infect Disord Drug Targets. 2012;12: 200–212. 22394174 10.2174/187152612800564419 PMC3420344

