# Supplementary material for: Entry of Francisella tularensis into Murine B Cells: The Role of B Cell Receptors and Complement Receptors
Source: PLoS One. 2015 Jul 10;10(7):e0132571. doi: 10.1371/journal.pone.0132571 (PMC4498600; doi:10.1371/journal.pone.0132571)
Supplement: S1 Fig — The volume of 500 μL of fresh murine sera (C) or 500 μL of murine heat-inactivated fresh sera (iC) as a control were added to 4 x 10^9^ bacteria, incubated at 36.8°C for 1 h, washed twice with pre-warmed PBS, resuspended in 1 mL saline, and used for the experiments. To opsonized bacteria the sera were added to 1 mL of bacterial suspension. A20 cells (5 x 10^5^ per well) were co-cultivated with unopsonized, opsonized with C, and iC resp. F. tularensis LVS/GFP in total volume of 0.5 mL per well at MOI 500. Control A20 cells were cultivated without infection. After 3 h incubation cultures were washed using PBS at 36.8°C and 5% CO2 atmosphere. The proportion of infected cells with F. tularensis LVS/GFP was examined using flow cytometry. Error bars indicate SD around the means of samples processed in triplicate. Relative infectivity was calculated as a ratio among A20 cells infected with F. tularensis LVS/GFP and A20 cells infected with opsonized bacteria. Proportional number of infected A20 cells with GFP was laid to be one. Two-tailed t-test was used to find significant difference between GFP and GFP+C and, GFP+iC (* P < 0.05). Results shown from one experiment are representative of three independent experiments. Note: Heat-inactivated sera were prepared by heating sera in water bath (56°C) at the volume of 0.5 mL for 30 min. (PDF) [file pone.0132571.s001.pdf]

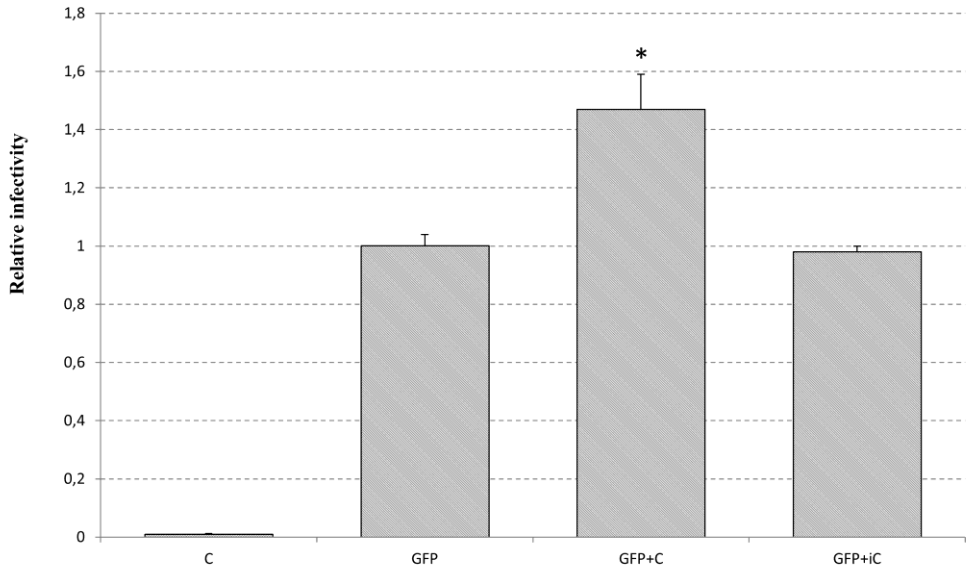

S2 Fig. Relative infectivity of *F. tularensis* LVS opsonized with fresh murine sera (C) and inactivated fresh murine sera (iC). The volume of 500 μL of fresh murine sera (C) or 500 μL of murine heat-inactivated fresh sera (iC) as a control were added to 4 x 10^9 bacteria, incubated at 36.8°C for 1 h, washed twice with pre-warmed PBS, resuspended in 1 mL saline, and used for the experiments. To opsonized bacteria the sera were added to 1 mL of bacterial suspension. A20 cells (5 x 10^5 per well) were co-cultivated with unopsonized, opsonized with C, and iC resp. *F. tularensis* LVS/GFP in total volume of 0.5 mL per well at MOI 500. Control A20 cells were cultivated without infection. After 3 h incubation cultures were washed using PBS at 36.8°C and 5% CO_2 atmosphere. The proportion of infected cells with *F. tularensis* LVS/GFP was examined using flow cytometry. Error bars indicate SD around the means of samples processed in triplicate. Relative infectivity was calculated as a ratio among A20 cells infected with *F. tularensis* LVS/GFP and A20 cells infected with opsonized bacteria. Proportional number of infected A20 cells with GFP was laid to be one. Two-tailed

t-test was used to find significant difference between GFP and GFP+C and, GFP+iC (* $P < 0.05$). Results shown from one experiment are representative of three independent experiments.

Note: Heat-inactivated sera were prepared by heating sera in water bath (56°C) at the volume of 0.5 mL for 30 min.
