# Supplementary material for: Entry of Francisella tularensis into Murine B Cells: The Role of B Cell Receptors and Complement Receptors
Source: PLoS One. 2015 Jul 10;10(7):e0132571. doi: 10.1371/journal.pone.0132571 (PMC4498600; doi:10.1371/journal.pone.0132571)
Supplement: S1 Table — After 24 h peritoneal cells were collected, resuspended in DMEM cultivation medium supplemented with 2% fetal bovine serum and then incubated with the antibody CD19-Alexa Fluor 647. Peritoneal CD19+ cells were sorted using BD FACSAria II Cell Sorter. Sorted CD19+ cells were washed using PBS and lysed by 0.1% sodium deoxycholate after washing. Actual numbers of bacteria were determined by serial dilutions (100 and 10−2) and the number of CFU was calculated. 1 Number of CD19+ cells seeded onto McLeod plates in volume 50 μL and cultivated at 36.8°C. 2 The number of CFU was determined after 48–72 h of cultivation. (PDF) [file pone.0132571.s002.pdf]

S1 Table. Viability of bacteria inside the B cells.

Mouse No.	Number of cells[1]	Number of CFU[2]
**1**	2.3×10^4	1.05×10^2
**2**	4.1×10^4	1.62×10^2
**3**	4.8×10^4	2.13×10^2

BALB/c mice were infected with *F. tularensis* LVS/GFP. After 24 h peritoneal cells were collected, resuspended in DMEM cultivation medium supplemented with 2% fetal bovine serum and then incubated with the antibody CD19-Alexa Fluor 647. Peritoneal $CD19^+$ cells were sorted using BD FACSAria II Cell Sorter. Sorted $CD19^+$ cells were washed using PBS and lysed by 0.1% sodium deoxycholate after washing. Actual numbers of bacteria were determined by serial dilutions (10^0 and 10^{-2}) and the number of CFU was calculated.

[1] Number of $CD19^+$ cells seeded onto McLeod plates in volume 50 μL and cultivated at 36.8°C.

[2] The number of CFU was determined after 48-72 h of cultivation.
